# Awake surgery for lesions near eloquent brain under scalp block and clinical monitoring: experience of single center with limited resources

**DOI:** 10.1186/s41983-021-00333-0

**Published:** 2021-06-15

**Authors:** Esam Abdelhameed, Mohamed Shebl Abdelghany, Hazem Abdelkhalek, Hytham Ibrahim Shokry Elatrozy

**Affiliations:** 1 Department of Neurosurgery, Faculty of Medicine, Tanta University, Tanta, Egypt; 2 Department of Anaethesia and Surgical Intensive Care, Faculty of Medicine, Tanta University, Tanta, Egypt; 3 Department of Neuropsychiatry, Faculty of Medicine, Tanta University, Tanta, Egypt; 4 Neurosurgery Department, Tanta University Hospital, Elgeish Street, Tanta, 31257 Egypt

**Keywords:** Awake craniotomy, Brain gliomas, Eloquent areas, Scalp block

## Abstract

**Background:**

Surgery of the brain tumors near eloquent areas carries the risk of either disabling neurological deficit or inadequate resection with bad prognosis in both situations. Awake surgery is the gold standard procedure for such lesions. However, it requires certain anesthetic drugs, advanced techniques, and trained teams that are not available in every neurosurgical institute. This work aims to evaluate safety, feasibility, and outcome of operating on patients with space occupying lesions near eloquent areas under scalp block being continuously examined by a neurologist through retrospective study of 20 cases with supratentorial lesions related to language or sensorimotor cortex.

**Results:**

There were 12 males and 8 females with mean age 36.8 years. Forty percent of patients were presented by motor weakness. Tumors were related to motor cortex in 11 patients and to language areas in 9 patients. Mean operative time was 210 min. Gross or near total resection was achieved in 15cases, four cases had subtotal resection and biopsy only was done in 1 case. Two patients suffered from intraoperative seizures and conversion to general anesthesia was required in one patient.

**Conclusion:**

Operating on tumors near eloquent brain areas under scalp block and continuous neurological examination during tumor resection proved to be effective in early detection and prevention of permanent major deficits especially in the developing countries with limited resources.

## Background

Surgery of brain tumors near eloquent areas may carry risk of neurological deficit or at least may limit the amount of tumor resection with bad prognostic effect in both situations [1]. The idea to operate on brain tumors under local anesthesia came from knowing that the brain is a nonsensitive structure in spite of controlling the whole process of pain sensation. Also, the functional brain areas are not anatomically the same in all people and can be displaced by tumors or surrounding edema. This augmented the need of determining these eloquent areas and their relation to brain tumors case by case in order to have safe and effective surgery [2, 3].

New MRI modalities like DTI (diffusion tensor imaging) tractography, BOLD (blood oxygen level dependent) signal changes, and magnetoencephalography can be used preoperatively to determine eloquent brain areas and their relation to brain tumors [4–6]. However, surgery depending on preoperative localization of eloquent areas alone carries significant morbidity [7].

The idea of intraoperative cortical mapping started when Dr. Bartholow used Faradic current over the cortex and noticed contralateral movement but unfortunately his patient died from seizures. Later, Broadmann and Von Economo could make maps of the cerebral cortex. Penfield could develop many types of stimulators to safely stimulate the motor, sensory, auditory, visual, and speech centers. That could make revolution in surgery of tumors and vascular malformations near eloquent brain as well as epilepsy surgery [8].

The use of remifentanil and dexamedetomedine in neuroanesthesia and different techniques of neurophysiological monitoring and brain mapping to identify eloquent cortical and subcortical centers have been reported in literature. However, these anesthetic drugs, advanced techniques and trained teams are not available in every neurosurgical institute especially in the developing countries [9, 10].

The aim of this study is to evaluate safety, feasibility, and outcomes of operating on patients with space occupying lesions near eloquent areas under scalp block with local anesthesia and continuous neurological examination by a neurologist.

## Methods

This series includes a retrospective study of 20 cases; twelve males and 8 females operated between January 2015 and June 2018. All patients had supratentorial lesions related to language or motor cortex. The ethical approval of this study was obtained from the research ethics committee. Consent of publication was obtained from patients whose photos or radiology images are found in the paper.

Absolute contraindications to awake surgery in our institute include patients with infratentorial lesions, uncooperative patients (children and psychiatric patients), and morbid obese patients with expected obstructive sleep apnea. Relative contraindications included chronic cough, nausea, patients with anticipated difficult airway, patients with uncontrolled seizures, patients with severe dysphasia (more than 25% naming errors), and those with motor weakness who cannot move their limbs against gravity especially if they did not improve after dehydrating measures.

### Preoperative preparation

All patients were subjected to detailed neurological examination, airway assessment, and routine lab investigations.

Brain CT (Aquilion One 320 slice, Toshiba America Medical System, Tustin, USA), MRI with contrast, and MRI tractography (Vantage Titan 1.5 T, Toshiba America Medical Systems, Tustin, USA) were done in all cases to clearly demarcate the lesion and its relation to eloquent brain. Functional MRI with BOLD technique was done in some cases to better localize eloquent cortex.

Patients’ data and images were carefully studied and discussed between the surgeon, anesthesiologist, and neurologist. The specific requirements in each case were fulfilled individually. All the OR team including the nurses and technicians were prepared to work in a very quiet environment.

Once the decision to operate under scalp block was made, informed consent was taken in all operations declaring the procedures advantages, difficulties, and alternatives. Some patients were shown operative videos to be psychologically prepared to their mission. Baseline sensorimotor and language assessment including naming, reading, spelling, and calculation was performed 24–48 h before surgery and the test was modified to exclude difficult tasks for the patient to be avoided during intraoperative monitoring.

Preoperative medications in the form of steroids (dexamethasone 8 mg), benzodiazepine (bromazepam 3 mg), and antiepileptic (phenytoin 500 mg) were given to the patient the night before operation. Antibiotics (ceftriaxone 1 g) and antiemetic (ondansetron 8 mg) were given 1 h before skin incision.

### Operative technique

All cases were operated under scalp block using local anesthetic throughout the whole procedure. In our center, ultrashort acting opioids such as remifentanil and dexmedetomidine were not available at the time of study. Alternative short acting opioids such as propofol (75–250 μg/kg/min) and fentanyl (.5-1 μg/kg/h) were given by the anesthesiologist when highly mandatory as they should to be stopped 20 min before testing.

**Fig. 1:**
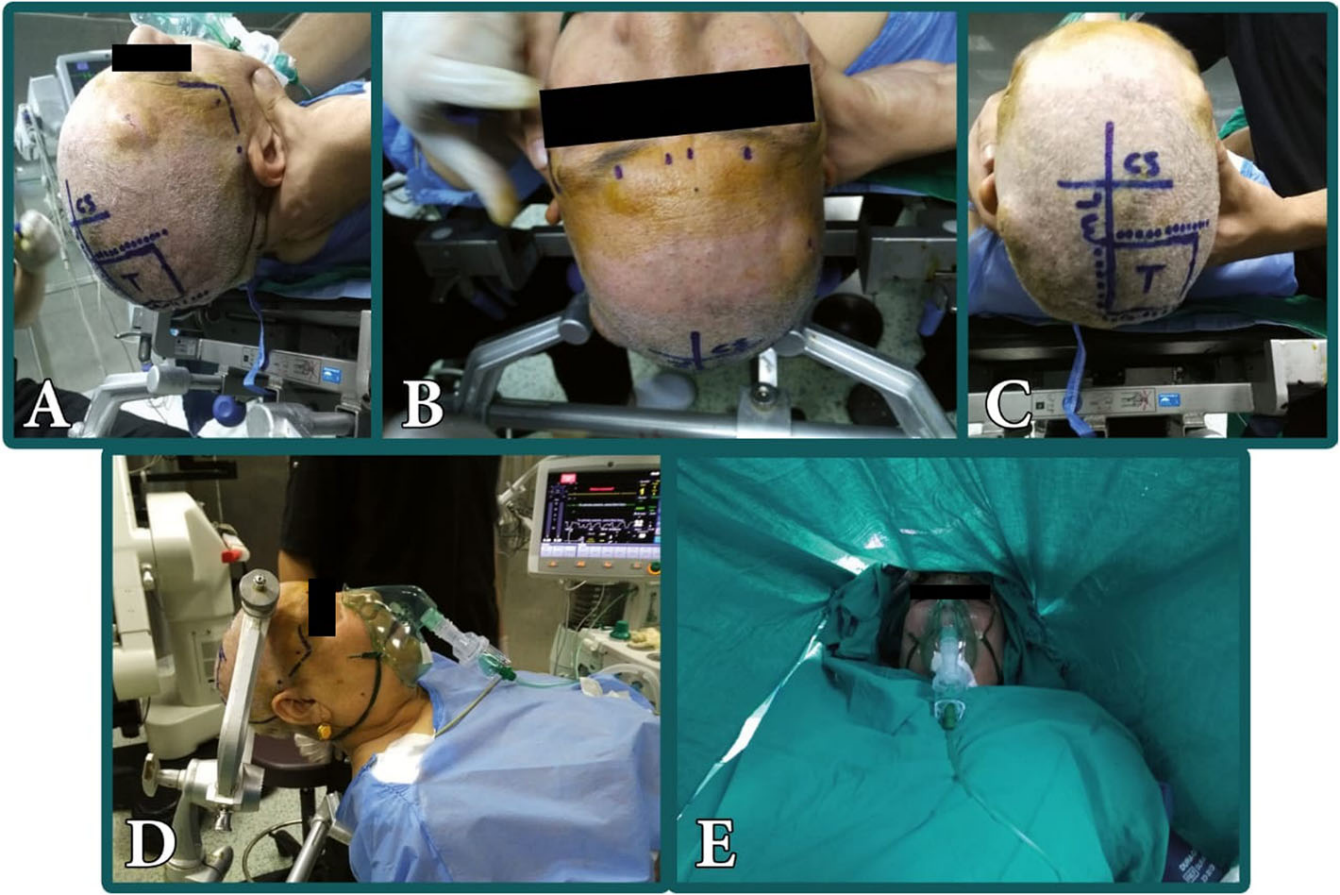
Patient positioning and scalp blockade with local anesthetic. **A**, **B** Sites of scalp infiltration with local anesthetic. **C** Tumor localization and designed scalp incision (T, tumor; ML, midline; and CS, coronal suture dotted line represents planned skin incision). **D** Application of three-pin head fixation. **E** Surgical draping permit free communication with the patient

The tumor location was calculated from patient images and marked on the scalp and the related functional brain areas were also marked and their relation to the tumor was studied carefully. The site of the planned skin incision was marked (Fig. 1).

A local anesthetic mixture of 30 ml of 0.25% bupivacaine and 0.5% lidocaine with 20 ml saline adrenaline (1:200,000) and 23-G needle were used for scalp block. The supraorbital notch was firstly injected (supraorbital nerve block) then the needle was redirected medially to block the supratrochlear nerve. The zygomaticotemporal nerve was blocked 1 cm lateral to the outer canthus and 1 cm above the zygomatic arch; then, the auriculotemporal nerve was blocked just above the auricle and beside the parietal branch of superficial temporal artery, we do not inject at the level of the tragus to avoid facial anesthesia. The greater auricular nerve was blocked posterior to the ear at the level of the tragus. Lastly, the greater and lesser occipital nerves were blocked along the superior nuchal line anterior and posterior to the occipital artery. The same steps were repeated on the contralateral side. We also injected local anesthesia at the site of planned skin incision (Fig. 1).

**Fig. 2:**
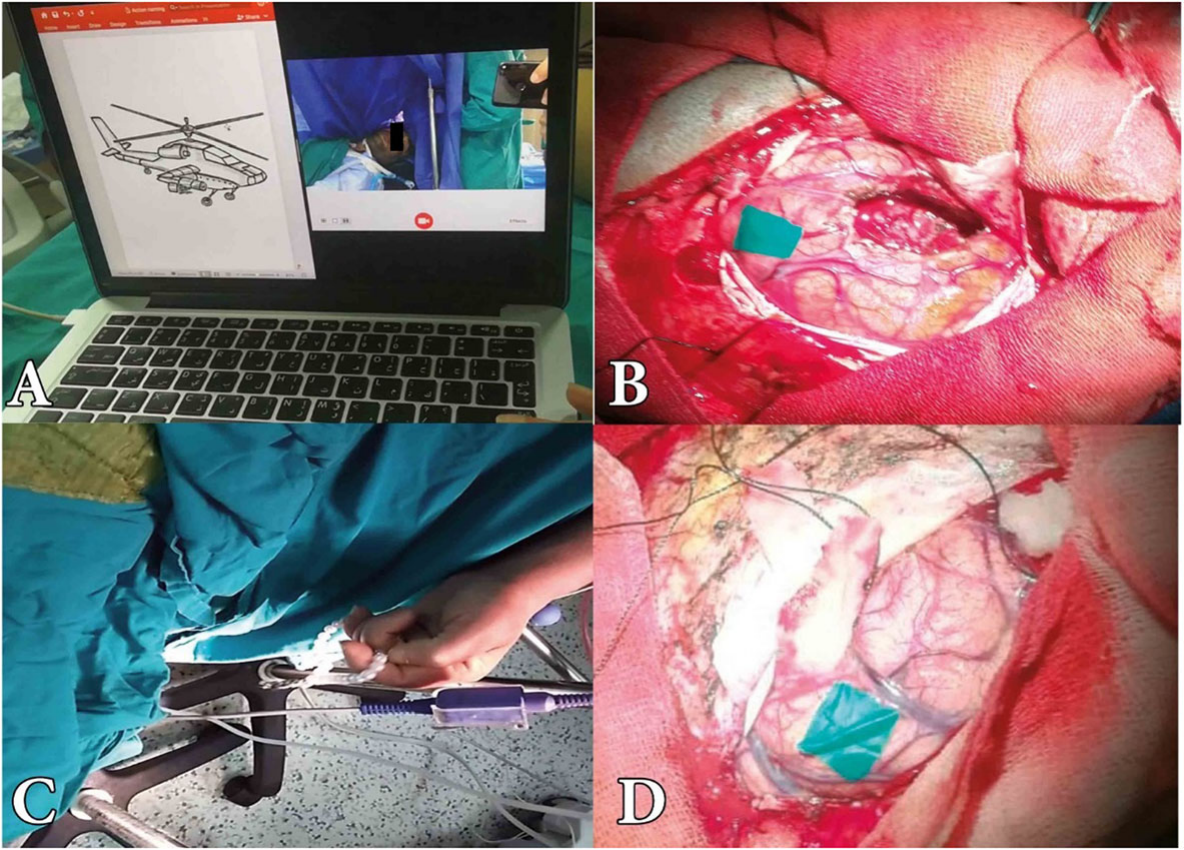
Intraoperative mapping of eloquent areas. **A** The patient has easy visual access to computer screen projecting images and record sound for speech monitoring. **B** Speech area identified by a piece of colored glove and tumor resection performed more than 1 cm away from language area. **C** Contralateral limb exposed and patient continuously manipulate rosary for motor monitoring. **D** Motor area identified after cortical stimulation

The three-pin head frame (Mayfield 2000, Integra™, Cincinnati, USA) was prepared and sites of head ring pins were marked and infiltrated with local anesthesia. Patient positioning was fashioned according to the tumor location. In all cases, the patient should have comfortable position with head elevated 20° above the heart level. Surgical draping was fashioned to allow continuous direct access between the anesthesiologist and the patient (Fig. 1). The contralateral extremity should be exposed for motor evaluation and the patient should have easy visual axis to a computer screen projecting images for speech monitoring (Fig. 2).

The patient was kept warmed and covered by a blanket to avoid shivering and O_2_ delivered via nasal cannula or face mask. Patient monitoring by neuroanesthesia was made by noninvasive blood pressure recording, cardiac monitors, pulse oximetry, end expiratory CO_2_ concentration, temperature, and urine output.

Skin incision and craniotomy were made in the ordinary manner. The dura was infiltrated with local anesthesia with an insulin syringe at the site of durotomy. Before durotomy, the patient was asked to take multiple deep breaths (hyperventilation) to decrease the intracranial pressure and at the same time to check if the patient is cooperative. Extra dose of manitol was given if needed.

In our center, we had neither intraoperative neurophysiological monitoring nor trained neurophysiologist; we could not perform motor evoked potential, electromyography, or electrocorticography. So we depended on direct electrical stimulation using bipolar stimulator (ISIS, Inomed, Emmendingen, Germany) delivering biphasic waves in 4 s trains at 60 HZ at low current < 5 mA .We started stimulation at 1.5 mA and increase at 0.5 m increments each time and watched for contralateral limb movement, speech arrest during counting from 1 to 30, anomia or alexia of projected images and words, respectively. We did not stimulate above 5 mA to avoid seizures. If mapping was positive for eloquent cortex, we would mark this area with a piece of colored gloves or cottonoid and keep surgery at least 1 cm away from it (Fig. 2). If no response was encountered till 5 mA, we would consider mapping negative. We use the same stimulation technique for suspected subcortical eloquent areas.

Microscopic surgery under highest possible magnification (Leica 525 OH4, Leica Microsystems, Wetzlar, Germany) was used to help clear demarcation of tumor tissue and its differentiation from normal brain tissue. The site of cortical incision was determined after neuroimages revision. Natural transsulcual corridors were followed when feasible to minimize cortical trauma.

The patient must be fully conscious, alert, and continuously examined by the neurologist till the tumor resection is finished and hemostasis completed. Language was assessed by asking the patient to count from 1 to 30 or speak letters of alphabets and waiting for points of speech arrest. Also by naming objects, or reading and waiting for points of anomia or alexia. Motor power was assessed by asking the patient to perform simple movements. It can also be continuously monitored by asking the patient to manipulate a rosary in his hand and watching for movement cessation or falling of the rosary from the patient hand (Fig. 2).

If any early neurological affection was noticed, manipulation was stopped, an extradose of steroid was given, and irrigation with warm saline was done waiting till neurological deficit disappears. The suspected area is examined by direct electrical stimulation using the same technique mentioned before, and then we could continue the procedure. If intraoperative seizures occurred, we stop brain manipulation and irrigate with cold ringer till fits stop.

After tumor resection, careful hemostasis was done and the patient was examined again to exclude any neurological deficit. Wound was closed and the Mayfield was removed and the patient was examined one more time before leaving the OR.

**Fig. 3:**
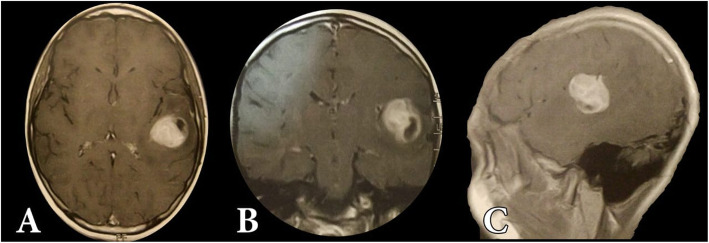
Preoperative axial (**A**), coronal (**B**), and sagittal (**C**) MRI with gadolinium images of a 14-year-old female patient presented with seizures, showing lt sided sol related to Wernick’s area. She was operated under local anesthesia and went home 2 days after surgery

**Fig. 4:**
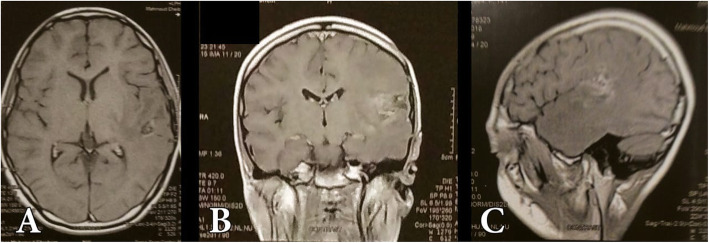
MRI with gadolinium follow-up of the same patient showing total resection of the tumor, pathology revealed astrocytoma grade III

**Fig. 5:**
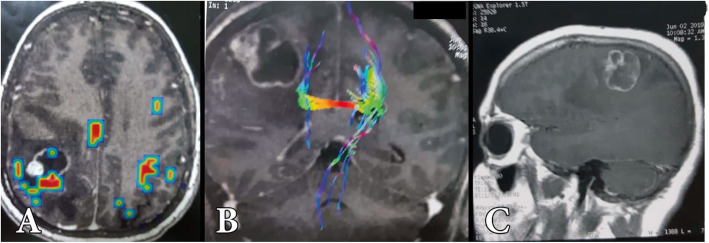
Preoperative axial bold (**A**), coronal tractography (**B**), and sagittal post-contrast MRI images of a 32-year-old female patient presented by seizures and hemiparesis grade 4 history of cancer ovary with oopherectomy and hysterectomy since 2 years

**Fig. 6:**
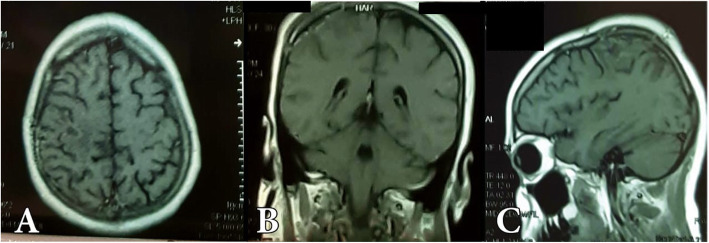
Postoperative MRI with gadolinium of the same patient in axial (**A**), coronal (**B**), and sagittal (**C**) projections showing total tumor resection

Follow-up CT brain with contrast was routinely asked the next day and the patient could go home 48 h after surgery. Follow-up MRI brain with contrast was performed within 3 months later (Figs. 3, 4, 5, and 6).

## Results

This study included 20 patients with tumors near eloquent brain areas subjected to surgery under local anesthesia between January 2015 and June 2018. There were 12 males (60%) and 8 females (40%). Their age ranged between 14 and 62 years with mean age was 36.8 ± 14.76 years.

Motor weakness was the most common clinical presentation encountered in 8 patients (40%). Six patients had motor power grade IV and 2 patients had motor power grade III on MRC grading system. Patients with motor power grade II or less were not candidates as they were not amenable to continuous examination by the neurologist. Distributions of different clinical presentations were summarized in Table 1.

**Table 1:** Distribution of clinical presentation

Clinical picture	n	Percent
Headache	6	30
Motor weakness	8	40
Grade IV	6	30
Grade III	2	10
Seizures	6	30
Speech difficulty	3	15

All tumors were related to eloquent brain; 11 tumors were related to motor cortex, and 9 tumors were related to language area (Table 2).

**Table 2:** Relation of the tumor to eloquent cortex

Tumor location	n	
Motor cortex rt	6	30%
Motor cortex lt	5	25%
Broca’s area	4	20%
Wernike’s area	5	25%

After stimulation mapping was positive for eloquent cortex in 16 patients. Four patients showed negative mapping, and all of them had lesions related to speech areas. Subcortical stimulation was positive in 12 patients.

All patients were operated under local anesthesia with scalp block. However, conversion to general anesthesia was needed in one patient due to uncontrolled seizures.

Unfavorable intraoperative events included pain during temporalis muscle dissection occurred in two patients managed by giving fentanyl and sedation. Two patients had intraoperative seizures that stopped in one of them and the procedure could be completed normally but the other case developed serial fits and conversion to general anesthesia was mandatory. This patient needed postoperative mechanical ventilation and died 2 weeks later from ventilator associated pneumonia.

Operative time ranged from 170 to 250 min with mean operative time 210 ± 25.33 min. Hospital stay ranged from 2 to 14 days with the mean hospital stay 2.7 ± 2.67 days.

### Radiological outcome

Most of tumors underwent gross or near total resection (15 cases); four cases had subtotal resection with tumor residual more than 1 cm and biopsy only was done in 1 case.

Glioblastoma multiform grade IV was the most frequent tumor pathology encountered in 10 cases. Six of them were related to motor area and 4 to speech area. Table 3 summarizes different pathologies encountered in this study.

**Table 3:** Histopathological diagnosis

Tumor pathology	n	
Glioblastoma IV	10	50%
Anaplastic astrocytoma III	5	25%
Oligoastrocytoma	3	15%
Pilocytic astrocytoma	1	5%
Metastatic adenocarcinoma (ovary)	1	5%

Gross total resection was achieved in 100% of metastatic adenocarcinoma (one case), pilocytic astrocytoma (one case), and anaplastic astrocytoma (5 cases). We had 3 cases of oligoastrocytoma; two of them underwent gross total resection and biopsy only was done in one case due to uncontrolled intraoperative seizures and conversion to general anesthesia with fear from neurological deficit. Gross total resection was achieved in 60% and subtotal resection in 40% of glioblastoma multiform cases.

### Clinical outcome

One case developed postoperative seizures and follow-up CT brain showed non-surgical intracerebral hematoma in tumor bed that was absorbed spontaneously.

Eight patients developed postoperative neurological deterioration. One patient suffered from increased dysphasia after surgery and improved completely within 1 month. Seven patients experienced postoperative deterioration of motor power. One of them had normal motor power preoperatively and developed weakness following intraoperative seizures that improved totally on the second day postoperatively (postictal Todd’s paralysis). The remaining six patients were known to have preoperative weakness. Motor deterioration improved in all cases within 2 months except one case had persistent spastic hemiparesis.

## Discussion

The main goal in brain tumor surgery is maximal or total resection with minimal or no neurological deficit. This gave special concern to tumors related to eloquent brain [1]. Many trials have been made to localize the eloquent brain areas either preoperatively by advanced functional MRI techniques as DTI, BOLD, and magnetoencephalography or intraoperatively by the use of neuronavigator [4–6, 11]. However, safe surgery cannot depend on these methods alone [7, 12].

Awake craniotomy with intraoperative mapping is the gold standard technique for safe and effective surgery of the brain eloquent areas. The technological demands of neurophysiological monitoring such as evoked potentials, EMG, and EEG and trained neurophysiologists in addition to certain new anesthetic drugs such as remifentanil and dexmedetomidine are not available in every neurosurgical center especially in developing countries. Many reports described the early experience of awake surgery in localities with limited resources [13–17]. In this study, we describe our experience with awake surgery in our institute. Patients with tumors near the brain eloquent areas were operated under local anesthesia with continuous examination during surgery by a neurologist.

Different anesthesia techniques were described in relation to awake craniotomy. These techniques include asleep-awake-asleep technique where patient is awake only during examination and mapping and airway should be protected. Another technique is the monitored anesthesia control or conscious sedation where the patient is awake but under the effect of mild sedation, so he can cooperate with mapping tasks. The third technique is known as awake-awake-awake technique where the patient is under the effect of local or regional anesthesia and intravenous analgesia [18–20]. In this study, we depend on scalp block with long-acting local anesthetics which enable application of the three-pin head holder in the way that maintains patient comfortability. Extradoses of local anesthesia were given with skin and dural incisions. This allows the patient to be fully conscious and highly cooperative with the neurologist during examination and provides no need of airway protection. The use of sedation in the form of intravenous propofol plus fentanyl was reserved to mandatory situations (two patients due to irritability caused by pain related to temporalis dissection, and one patient with intraoperative fits not stopped by cold ringer irrigation) as it may hinder continuous examination for more than 20 min.

Hervey-Jumper and colleagues reported that the incidence of intraoperative seizures in literature varies from 2.2 to 21.5%. In their own series, intraoperative seizures were encountered in 3% of patients (20/611) and stopped by cold ringer irrigation in all cases except for 2 patients. That mandates to stop the procedure. They reported that the presence of preoperative seizures and tumor location is related to the occurrence of postoperative seizures [21]. In this study, intraoperative seizures occurred in two patients (10%) and necessitate procedure abortion in one of them. Both patients had preoperative seizures. The tumor was related to motor cortex in both patients. Both patients were operated early in our study where relatively higher intensity and more frequent stimulation were used. We think that higher incidence of intraoperative seizures in our series of 20 patients in comparison to Hervey-Jumper series of 611 patients can be attributed to our small sample. Leal RTM and colleagues’ series of 19 patients reported 4 cases of intraoperative seizures (21%) [15].

Negative mapping does not guarantee absence of eloquent sites as it may be due to methodological reasons. When mapping is negative, the dilemma is either to operate at the stimulated area with fear from neurological deficit or to increase stimulation intensity with fear from intraoperative seizures. Sanai and colleagues performed small craniotomies depending on negative mapping for resection of language area related tumors and they reported 4 cases of permanent language deficit in the surviving patients [22]. Other authors recommend wider bone flaps to achieve positive mapping which provide safer and better extent of resection [23]. Advanced neurophysiological monitoring techniques such as evoked potentials and EMG can detect eloquent cortex earlier at low-intensity currents but we do not have them, so we depended on making larger bone flaps to have sufficient area for stimulation and decrease the incidence of false negative mapping. We avoided frequent high-intensity stimulation to decrease the incidence of seizures. If mapping is negative at 5 mA, we do not increase intensity. We had negative mapping in only 4 patients. Surprisingly, Nossek and colleagues found an increased incidence of seizures when intraoperative EEG was used. That may be explained by the false sensation of safety to use higher intensity current stimulation [24].

To avoid patient intolerance, we recommend taking more time in preoperative preparation and spending more efforts in patient education to be psychologically prepared. We do not recommend changing the awake craniotomy team. The surgeon, the assistant, the nurse, the anesthesia technician, the neuroanesthesiologist, the neurologist, and the operating room were fixed in all cases. The only changeable factor in the awake craniotomy should be the patient, to increase learning curve and team harmony and to minimize operative time. The mean operative time in our series (210 min) was approximately equal to that of Joswig et al. [14] (205 min), but it is shorter than Tze-Ching et al. [25] (258 min). This may be attributed to time spent in neurophysiological monitoring techniques.

In this series, conversion to general anesthesia was required in 1 case (5%) due to uncontrolled seizure. The patient underwent only a biopsy to avoid occurrence of neurological deficits; Hervey-Jumper and colleagues aborted the procedure and converted to general anesthesia in 3 cases (0.5%) due to seizures in two cases and emotional intolerance in one case [21].

Extent of tumor resection and postoperative neurological deficit are two conversely related goals. Rahman and colleagues reported occurrence of 30% new postoperative deficit associated with 94.2% mean extent of operative resection. These deficits improved in 41% on further follow-up [26]. Shinoura and colleagues reported that preoperative weakness, close proximity or compression of motor tract, and location within premotor area were associated with worsening of neurological deficit [27]. Transient neurological deficit was reported in the literature in eloquent brain surgery and was attributed mainly to brain edema at resection margins; delayed improvement was recorded in most of the cases after resolving of brain edema. Duffau and colleagues reported increased incidence of postoperative deficit ranged from 13 to 27.5% without the use of awake mapping. On the other hand, the use of awake mapping is associated with a permanent deficit rate less than 2% [28]. With the patient under local anesthesia intraoperative neurological deficit can be detected early and reversed by temporary cessation of brain manipulation which will prevent or at least minimize postoperative neurological deficit. Transient neurological deficit was observed in 8 cases (40%); follow-up CT brain showed brain edema at resection margins in all cases. The deficit resolved completely within 2 months in 7 cases. Mapping was positive in all cases and this match with later improvement of neurological deficits and can be attributed to transient brain edema. One case in our series (5%) had permanent deficit. This was similar to the results of Tze-Ching et al. [25] who reported one case of permanent neurological deficit in their series of 21 patients (4.2%); it was a recurrent tumor. Taylor and Bernstein reported higher incidence of permanent deficit in redo craniotomies (12%) compared to de novo craniotomies (2.5%) [29]. Hervey-Jumper and colleagues in their larger series reported 9% incidence of early neurological deficit that reduced to 3% after 3 months [21].

The main limitations of this study are the retrospective nature, small number of cases, and absence of second arm comparing this procedure to conventional craniotomy under general anesthesia.

## Conclusion

Operating on tumors near eloquent brain areas under scalp block with continuous neurological examination during tumor resection has proven to be good in early detection of neurological deficit and prevention of permanent major deficits especially in the developing countries with limited resources.

## Data Availability

The datasets used and/or analyzed during the current study are available from the corresponding author on reasonable request.

## Abbreviations

BOLD: Blood oxygen level dependent
CT: Computerized tomography
DTI: Diffusion tensor imaging
EMG: Electromyography
EEG: Electroencephalography
MRI: Magnetic resonance imaging
OR: Operating room
